# Crossing the road in time: Inequalities in older people's walking speeds

**DOI:** 10.1016/j.jth.2017.02.009

**Published:** 2017-06

**Authors:** Elizabeth A. Webb, Steven Bell, Rebecca E. Lacey, Jessica G. Abell

**Affiliations:** a Research Department of Epidemiology and Public Health, University College London, London UK; b Department of Public Health and Primary Care, University of Cambridge, Cambridge, UK; c Centre for Research in Epidemiology and Population Health, INSERM, Unit 1018, Villejuif, France

## Abstract

Pedestrian crossings in the UK and US require people to walk at 1.2 m/s to cross the road in time; however a large proportion of older people do not walk this fast, potentially discouraging walking or putting older people at risk of injury. We use longitudinal data to investigate changes in walking speed, and ability to cross the road in time, at older ages.

31,015 walking speed measurements were taken from 10,249 men and women aged 60+ years in waves 1–7 of the English Longitudinal Study of Ageing (2002–2014). Growth curve analyses were used to model how walking speed changes with increasing age, and predicted probabilities of being able to cross the road in time were estimated.

10% of measured walking speeds were fast enough to cross the road in time. Walking speed declined with age (−5.7×10^−3^m/s/yr (95% CI −7.6×10^−3^, −3.9×10^−3^)), and the decline accelerated with increasing age (−0.3 ×10^−3^m/s/yr (−0.4 ×10^−3^, −0.3 ×10^−3^)). Female, less wealthy and less healthy older people had slower walking speeds. For instance, predicted probability of crossing the road in time at age 60 was 14.8% (10.1, 18.5) and 2.7% (1.5, 3.8) for the richest and poorest men and 8.4% (6.0, 1.1) and 1.5% (0.9, 2.2) for the richest and poorest women, and at age 80 they were 7.1% (3.6, 10.5) and 1.0% (0.3, 1.7) for the richest and poorest men and 3.7% (1.6, 5.9) and 0.5% (0.1, 0.9) for the richest and poorest women.

Most older people do not walk fast enough to cross the road in time. Even the majority of the wealthiest and healthiest people aged 60 years and older do not walk fast enough to cross pedestrian crossings in the allocated time. Crossing times should be increased to allow for older peoples’ slower walking speeds or other policies considered to improve walkability, and to help avoid injuries and social isolation.

## 1 Introduction

Pedestrian crossings in the UK and US require people to walk at 1.2 m/s to cross in time (Pedestrian Facilities at Signal-Controlled Junctions, 2005; Fitzpatrick et al., 2006), however a large proportion of older people cannot walk at this speed (Asher et al., 2012; Zaninotto et al., 2013; Brunner et al., 2009; Musselwhite, 2014): existing cross-sectional evidence suggests that only about 20% of those aged 65+ years walk this fast (Asher et al., 2012). An inability to walk fast enough to cross the road safely may put older pedestrians at risk of death or injury in a road traffic accident and may inhibit older people's activities, for example, the fear of falling can lead to risk avoidance (Zijlstra et al., 2007). If older people are inhibited from walking, their access to amenities will be reduced, and risks of physical inactivity and social isolation will be increased, with attendant implications for healthy ageing (Shankar et al., 2011).

Older people report a preference for using designated pedestrian crossings, but also that they are given inadequate time to cross (Shaw et al., 2002). Cross-sectional studies have identified that women, those aged 75+ years, living in more deprived areas, who smoked or were in poor health had slower walking speeds (Asher et al., 2012, Brunner et al., 2009, Musselwhite, 2014), but have not assessed how individual's walking speeds change over time. Those few longitudinal analyses which have addressed this have identified wealth and educational differentials in walking speed, and declines in walking speed with age (Zaninotto et al., 2013, Weber et al., 2016) but have not investigated older people's changing ability to cross the road safely.

Our study extends the existing literature by investigating changes over time in both older people's walking speeds and older people's ability to cross the road in time, using longitudinal data collected over 12 years. We will estimate predicted mean walking speeds and predicted probabilities of the ability to cross the road in time and will interpret our findings with reference to the current required walking speed at pedestrian crossings.

## 2 Methods

### 2.1 Study sample and measures

This study used data from seven waves (2002–2014) of the English Longitudinal Study of Ageing (ELSA) (Steptoe et al., 2013), a longitudinal study of people aged 50+ years living in England. Participants gave full informed consent to participate in the study, and ethical approval was obtained from the National Research Ethics Committee. Timed walks were measured biennially in participants aged 60+ years. Participants walked eight feet (2.44 m) and back at their usual walking pace, using walking aids if required. Walking speed was calculated in metres per second (m/s), and we used the mean of the two measurements as our outcome variable. The eligible sample is 12,472 people, all of whom provided at least one walking speed measurement, resulting in 42,135 walking speed measurements over a 12 year follow up period. Our analytic sample included 10,249 individuals (54.7% female) who provided 31,015 walking speed measurements with complete data on all covariates.

Covariates, drawn from the literature (Asher et al., 2012, Zaninotto et al., 2013, Brunner et al., 2009), which we hypothesise will influence older people's walking speeds and ability to cross the road in time were: age (years, centred on age 60), age squared (to account for the non-linear change in walking speed with age), sex, wealth quintiles, smoking (never, former, current), limiting longstanding illness (LLSI), and difficulties with activities of daily living (Katz, 1963) (ADL; 0, 1 or ≥2 of a possible six), all of which were allowed to vary with time.

### 2.2 Statistical analysis

Growth curves models were fitted to estimate changes in walking speed with age. Covariates and covariate interactions with age and age squared were added in a stepwise fashion to determine whether they affected the intercept (estimated mean walking speed at age 60), slope (change in walking speed per year increase in age) or rate of change in the slope (change in walking speed per unit increase in the square of age in years). Only those covariates and covariate interactions with age and age squared which improved the model fit, assessed using likelihood ratio tests, were retained. All tested variables were significantly associated with the intercept; participant sex, wealth and difficulties with ADLs were associated with the slope and sex was furthermore associated with the rate of change in the slope. Predicted probabilities of being able to cross the road in time were calculated. All analyses were carried out in Stata 13.1.

## 3 Results

Only 10% of measured walking speeds were fast enough for the required pedestrian crossing speeds of 1.2 m/s. The ability to walk fast enough was more common amongst male (12% vs. 8% female), younger (18% at 60 years vs. 3% at 80 years), wealthier (20% of wealthiest quintile vs. 3% of least wealthy) and non-smoking (12% of never smokers vs. 6% or smokers) older people, and those without a LLSI (14% without vs. 5% with) and no ADL difficulties (13% without difficulties vs. 3% with 2+ difficulties).

**Fig. 1:**
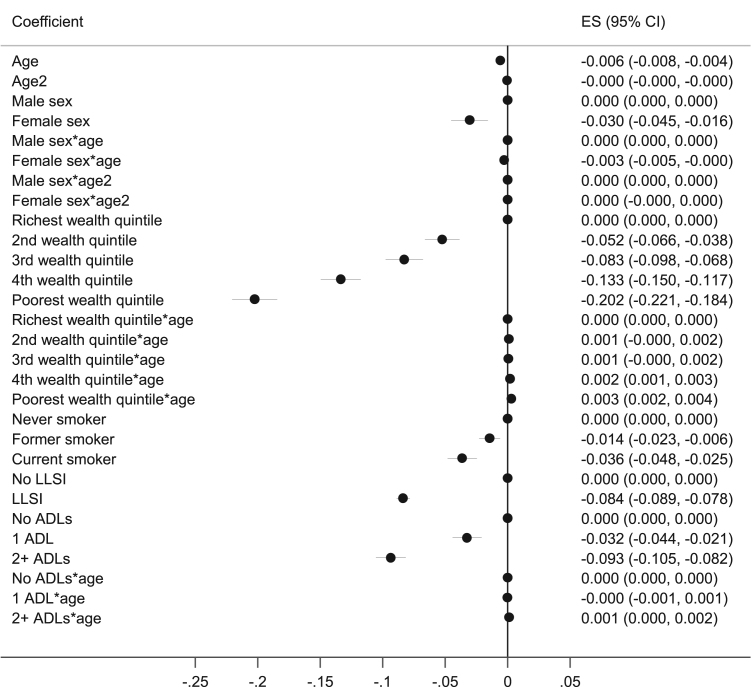
Forest plot showing coefficients and 95% confidence intervals from growth curve analysis.

**Fig. 2:**
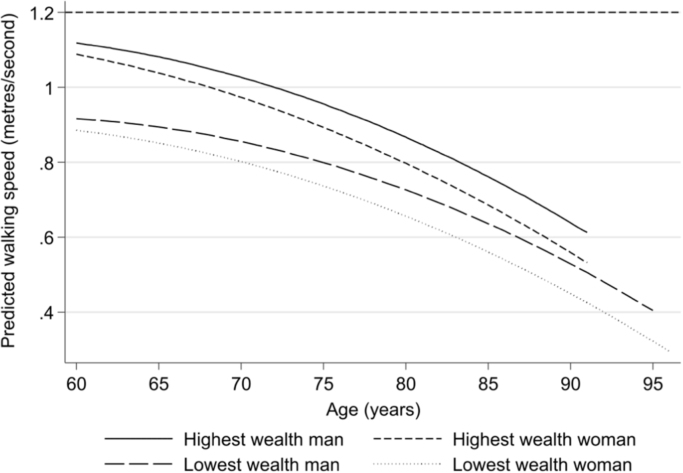
Predicted change in walking speed with age for men and women.^a^ The horizontal line at 1.2 m/s highlights the required walking speed to cross the road in time using pedestrian crossings in the UK and US. a. Predicted walking speeds for never smokers with no difficulties with ADLs and no limiting longstanding illness.

Coefficients and 95% confidence intervals for taken from growth curve modelling are shown in Fig. 1. This modelling predicted that mean walking speeds declined with age, and that this decline accelerated with advancing age. Walking speeds were slower for women, those with less wealth, former and particularly current smokers compared to never smokers, people with a LLSI compared to those without, and people with ≥1 compared to no ADL difficulties. Significant interactions with age indicated that women had a more rapid decline in walking speed than men, whilst those in the poorer wealth quintiles had a shallower decline in walking speed than those in the richer quintiles, and those with ADL difficulties had a shallower decline than those without ADL difficulties. Example predicted growth curves of walking speed with increasing age are shown in Fig. 2. These are for men and women in the highest wealth quintile, and mean and women in the lowest wealth quintile, who are never smokers and have no difficulties with ADLs or LLSI.

Predicted mean walking speeds and predicted probabilities of being able to cross the road in time, for older people in the different wealth quintiles, and smoking, ADL and LLSI categories are given, by age and sex, in Table 1. Predicted mean walking speeds and predicted probability of ability to cross the road in time were higher amongst younger people, men, wealthier people, non-smokers and those with no difficulties with ADLs or LLSI.

**Table 1:** Predicted mean walking speeds, by sex, age and covariates,* from growth curve analyses of waves 1 to 7 of the English Longitudinal Study of Ageing.

		60 years		70 years		80 years		90 years		100 years	
Walking speeds (m/s)		Predicted mean (95% CI)	Predicted % probability (95% CI)	Predicted mean (95% CI)	Predicted % probability (95% CI)	Predicted mean (95% CI)	Predicted % probability (95% CI)	Predicted mean (95% CI)	Predicted % probability (95% CI)	Predicted mean	Predicted % probability (95% CI)
Men											
											
Wealth quintile	Richest	1.00 (0.98, 1.02)	14.81 (10.10, 18.52)	0.95 (0.94, 0.96)	9.95 (8.37, 11.53)	0.90 (0.88, 0.91)	7.05 (3.63, 10.46)	0.84 (0.81, 0.88)	4.93 (0.64, 9.23)	0.79 (0.74, 0.84)	3.42 (−0.94, 7.78)
	2	0.95 (0.93, 0.97)	7.86 (5.47, 10.26)	0.90 (0.90, 0.91)	5.50 (4.49, 6.51)	0.86 (0.84, 0.88)	3.81 (1.77, 5.84)	0.82 (0.78, 0.85)	2.62 (0.15, 5.08)	0.77 (0.72, 0.83)	1.79 (−0.65, 4.23)
	3	0.91 (0.89, 0.94)	6.19 (4.21, 8.17)	0.87 (0.86, 0.88)	3.71 (2.95, 4.46)	0.83 (0.81, 0.84)	2.19 (0.95, 3.42)	0.78 (0.75, 0.82)	1.28 (0.02, 2.53)	0.74 (0.68, 0.79)	0.74 (−0.31, 1.80)
	4	0.87 (0.85, 0.89)	4.35 (2.81, 5.88)	0.83 (0.83, 0.84)	2.57 (1.97, 3.17)	0.80 (0.78, 0.82)	1.50 (0.59, 2.42)	0.76 (0.73, 0.80)	0.88 (−0.04, 1.80)	0.73 (0.68, 0.78)	0.51, −0.26, 1.27)
	Poorest	0.80 (0.77, 0.82)	2.67 (1.53, 3.80)	0.78 (0.77, 0.79)	1.66 (1.19, 2.13)	0.76 (0.74, 0.77)	1.03 (0.32, 1.74)	0.73 (0.70, 0.77)	0.64 (−0.11, 1.38)	0.71 (0.66, 0.77)	0.39 (−0.26, 1.05)
Smoking	Never	0.93 (0.91, 0.95)	9.62 (7.12, 12.12)	0.89 (0.88, 0.90)	6.61 (5.48, 7.76)	0.85 (0.83, 0.86)	4.50 (2.34, 6.67)	0.81 (0.77, 0.84)	3.05 (0.46, 5.64)	0.77 (0.72, 0.82)	2.06 (−0.48, 4.59)
	Former	0.91 (0.89, 0.93)	7.57 (5.60, 9.54)	0.87 (0.87, 0.88)	5.15 (4.32, 5.99)	0.83 (0.82, 0.85)	3.48 (1.79, 5.17)	0.79 (0.76, 0.82)	2.34 (0.33, 4.34)	0.75 (0.70, 0.80)	1.57 (−0.39, 3.52)
	Current	0.89 (0.87, 0.91)	5.33 (3.65, 7.01)	0.85 (0.84, 0.86)	3.58 (2.69, 4.47)	0.81 (0.79, 0.83)	2.39 (1.11, 3.68)	0.77 (0.73, 0.80)	1.60 (0.16, 3.03)	0.73 (0.68, 0.78)	1.06 (−0.30, 2.43)
											
LLSI	No	0.95 (0.93, 0.97)	9.19 (6.84, 11.26)	0.91 (0.90, 0.91)	6.28 (5.33, 7.21)	0.87 (0.85, 0.88)	4.25 (2.24, 6.26)	0.83 (0.79, 0.86)	2.86 (0.44, 5.27)	0.79 (0.74, 0.84)	1.92 (−0.45, 1.28)
	Yes	0.86 (0.84, 0.88)	4.84 (3.46, 6.23)	0.82 (0.82, 0.83)	3.22 (2.62, 3.84)	0.78 (0.77, 0.80)	2.15 (1.05, 3.24)	0.74 (0.71, 0.78)	1.42 (0.17, 2.68)	0.70 (0.65, 0.75)	0.95 (−0.25, 2.15)
											
ADL	0	0.93 (0.91, 0.95)	8.82 (6.54, 11.10)	0.89 (0.89, 0.90)	6.06 (5.15, 6.97)	0.85 (0.83, 0.87)	4.12 (2.16, 6.08)	0.81 (0.78, 0.84)	2.78 (0.42, 5.15)	0.77 (0.72, 0.82)	1.87 (−0.45, 4.19)
	1	0.90 (0.88, 0.92)	5.69 (3.71, 7.67)	0.86 (0.85, 0.87)	3.88 (3.06, 4.70)	0.81 (0.80, 0.83)	2.63 (1.10, 4.15)	0.77 (0.73, 0.80)	1.77 (−0.03, 3.58)	0.72 (0.67, 0.78)	1.20 (−0.57, 2.96)
	2+	0.84 (0.82, 0.86)	3.61 (2.15, 5.06)	0.81 (0.80,0.82)	2.02 (1.49, 2.55)	0.78 (0.76, 0.80)	1.13 (0.37, 1.88)	0.75 (0.71, 0.78)	0.62 (−0.10, 1.35)	0.72 (0.67, 0.77)	0.34 (−0.23, 0.92)

Women											
											
Wealth quintile	Richest	0.97 (0.95, 0.99)	8.42 (6.01, 1.08)	0.89 (0.88, 0.90)	5.64 (4.53, 6.75)	0.80 (0.79, 0.82)	3.73 (1.59, 5.86)	0.72 (0.69, 0.75)	2.44 (0.00, 4.88)	0.64 (0.59, 0.69)	1.58 (−0.70, 3.86)
	2	0.92 (0.90, 0.94)	4.63 (3.16, 6.12)	0.84 (0.84, 0.85)	3.03 (2.37, 3.69)	0.77 (0.75, 0.79)	1.97 (0.76, 3.17)	0.70 (0.67, 0.73)	1.27 (−0.07, 2.61)	0.62 (0.58, 0.67)	0.82 (−0.42, 2.05)
	3	0.89 (0.87, 0.91)	3.62 (2.41, 4.82)	0.81 (0.80, 0.82)	2.02 (1.54, 2.50)	0.74 (0.72, 0.75)	1.12 (0.40, 1.83)	0.66 (0.63, 0.69)	0.61 (−0.06, 1.29)	0.58 (0.54, 0.63)	0.34 (−0.19, 0.86)
	4	0.84 (0.82, 0.86)	2.52 (1.60, 3.43)	0.77 (0.77, 0.78)	1.39 (1.03, 1.76)	0.71 (0.69, 0.72)	0.77 (0.24, 1.29)	0.64 (0.61, 0.67)	4.19 (−0.07, 0.90)	0.58 (0.53, 0.63)	0.23 (−0.15, 0.61)
	Poorest	0.77 (0.75, 0.79)	1.53 (0.85, 2.20)	0.72 (0.71, 0.73)	0.89 (0.62, 1.17)	0.66 (0.65, 0.68)	0.52 (0.13, 0.91)	0.61 (0.58, 0.65)	0.30 (−0.08, 0.69)	0.56 (0.51, 0.61)	0.18 (−0.14, 0.49)
Smoking	Never	0.89 (0.87, 0.91)	5.38 (3.93, 6.83)	0.82 (0.81, 0.75)	3.43 (2.73, 4.14)	0.75 (0.73, 0.76)	2.17 (0.93, 3.42)	0.68 (0.65, 0.71)	1.37 (0.02, 2.72)	0.61 (0.56, 0.65)	0.86 (−0.35, 2.08)
	Former	0.87 (0.85, 0.89)	4.17 (3.02, 5.32)	0.80 (0.80, 0.81)	2.64 (2.07, 3.20)	0.73 (0.72, 0.75)	1.66 (0.69, 2.63)	0.66 (0.63, 0.69)	1.04 (0.00, 2.08)	0.59 (0.55, 0.64)	0.65 (−0.28, 1.58)
	Current	0.85 (0.83, 0.87)	2.89 (1.95, 3.82)	0.78 (0.77, 0.79)	1.81 (1.29, 2.33)	0.71 (0.69, 0.73)	1.13 (0.42, 1.84)	0.64 (0.61, 0.67)	0.70 (0.00, 1.43)	0.57 (0.53, 0.62)	0.44 (−0.20, 1.08)
											
LLSI	No	0.91 (0.89, 0.93)	5.43 (3.98, 6.89)	0.84 (0.84, 0.85)	3.45 (2.78, 4.12)	0.77 (0.76, 0.79)	2.17 (0.94, 3.41)	0.70 (0.67, 0.73)	1.37 (0.02, 2.71)	0.63 (0.59, 0.68)	0.86 (−0.35, 2.07)
	Yes	0.83 (0.81, 0.85)	2.78 (1.96, 3.60)	0.76 (0.75, 0.77)	1.73 (1.34, 2.12)	0.69 (0.68, 0.70)	1.08 (0.44, 1.72)	0.62 (0.59, 0.65)	0.67 (0.00, 1.35)	0.55 (0.51, 0.59)	0.42 (−0.18, 1.02)
											
ADL	0	0.90 (0.88, 0.92)	5.26 (3.84, 6.68)	0.83 (0.82, 0.84)	3.37 (2.72, 4.02)	0.76 (0.74, 0.77)	2.14 (0.92, 3.36)	0.69 (0.66, 0.72)	1.35 (0.02, 2.69)	0.61 (0.57, 0.66)	0.85 (−0.35, 2.06)
	1	0.87 (0.85, 0.89)	3.32 (2.14, 4.51)	0.80 (0.79, 0.80)	2.12 (1.59, 2.64)	0.72 (0.71, 0.74)	1.35 (0.46, 2.23)	0.65 (0.62, 0.68)	0.85 (−0.11, 1.81)	0.57 (0.52, 0.62)	0.54 (−0.33, 2.06)
	2+	0.81 (0.79, 0.83)	2.08 (1.23, 2.92)	0.75 (0.74, 0.76)	1.09 (0.78, 1.40)	0.69 (0.67, 0.70)	0.57 (0.15, 0.98)	0.63 (0.60, 0.66)	0.30 (−0.07, 0.66)	0.57 (0.52, 0.61)	0.15 (−0.12, 0.43)

^*^ All other covariates are held constant at the reference category (richest wealth quintile; never smoker; no LLSI; no difficulties with ADLs)

## 4 Discussion

Only 10% of walking speeds from our sample of people aged 60+ years were fast enough to cross the road in time at pedestrian crossings in the UK and US. The mean walking speed amongst even the youngest and most advantaged older people was too slow, with women, smokers, the less healthy and less wealthy old walking slower. Walking speeds declined with age and declined at a greater rate with increasing age, and the likelihood of being able to cross the road in time decreased with age.

Our results may, in fact, overestimate the proportion of older people able to cross the road in time. Firstly, our analyses only included the 74% of potential walking speeds which were measured: participants only completed the walking speed test if they were sufficiently healthy and mobile, and there was a safe setting in which to do so. Those for whom walking speed measurements were not available were more likely to be current or former smokers, to have difficulties with ADLs and to be in the poorer wealth quintiles, and are therefore likely to have slower walking speeds. Secondly, in the test participants were not carrying anything, whilst in a realistic setting they are likely to be carrying, for example, shopping, which would slow their walking speed further (Kong and Chua, 2014). Thirdly, our use of listwise deletion may have introduced selection bias, since those who remain in longitudinal studies tend to be healthier and more advantaged (Chatfield et al., 2005), and may therefore have faster walking speeds than those who drop out. This problem is partially ameliorated by our use of maximum likelihood estimation, so that participants who contributed walking speed measurements with complete data on covariates at least one wave are included in our models. Conversely, the measured walking speeds were participants’ usual, rather than maximum, walking speeds so some participants may be able to walk faster and cross the road in time.

Slow walking speeds and an inability to cross the road in time at older ages may increase the risk of social isolation, and wealth inequality in the ability to cros roads safely potentially has consequences for observed inequalities in social isolation (Jivraj et al., 2014). The least wealthy men and women had similar walking speeds at 60 years old to the wealthiest men and women 40 and 20 years older, respectively, and at all ages, men and women in the highest wealth quintile were more than five time more likely to be able to cross the road in time than their counterparts in the least wealthy quintile. These inequalities may be compounded by lower rates of car access amongst less wealthy older people (58% of the poorest quintile and 96% of the richest quintile), increasing reliance on walking.

Our findings reinforce existing cross-sectional work which finds that the majority of older people cannot cross the road in time (Asher et al., 2012, Musselwhite, 2014) although our estimates of the proportion able to walk fast enough are even lower. We support Asher and colleagues’ recommendation that crossing times be increased to allow for slower walking speeds (Asher et al., 2012). Other policies which may increase older people's confidence in crossing the road may be the use of ‘timed lights’, which count down the seconds remaining to cross (Shaw et al., 2012), or a policy like Singapore's innovative Green Man+ scheme, whereby older and disabled people receive a pass to tap on a reader at crossings, which then gives extra crossing time (Kong et al., 2013).

We conclude that the majority of older people are not able to cross the road in time and that there are substantial social inequalities in these abilities. Policy makers should consider implementing policies to make crossing the road easier and safer for older people.
